# Clinical Analysis and Optimization of Postremission Therapy for Acute Myeloid Leukemia Patients with Minimal Residual Disease as Determined by Flow Cytometry.

**DOI:** 10.4084/MJHID.2010.020

**Published:** 2010-08-05

**Authors:** Daichi Inoue, Hayato Maruoka, Takayuki Takahashi

**Affiliations:** 1 Department of Hematology and Clinical Immunology; 2 Clinical Laboratory, Kobe City Medical Center General Hospital, Kobe, Japan

## Abstract

**Background:**

Although several prognostic indicators of de novo acute myeloid leukemia (AML) patients have been identified, the clinical significance of minimal residual disease (MRD) needs to be evaluated further in Japanese adult patients.

**Methods:**

Using three color flow cytometry, we identified leukemia-associated phenotypes (LAP) in bone marrow specimens at diagnosis and assessed the relationship between clinical outcomes and the presence of marrow MRD in 33 patients who achieved a morphologic complete remission (CR) and were followed after CR.

**Results:**

Of 33 consecutive patients, we detected MRD in 20 patients after achieving CR (Group A) and did not in 13 patients (Group B), with 2-year overall survival (OS) rates of 49.0% and 84.6%, respectively (P =.0317), and relapse-free survival (RFS) rates of 13.7% and 91.7%, respectively (P=.0010). By multivariate analysis, MRD-positivity at post-induction was found to be associated with a shorter duration of RFS (P=.0042). Notably, we achieved MRD negativity in only 2 patients (10%) of Group A in spite of subsequent intensive consolidation therapies and found that the fluctuation of the MRD level during consolidation therapies was not a significant prognostic factor. Four patients in Group A underwent allogeneic hematopoietic stem-cell transplantation (HSCT) when in the CR state and did not experience relapse at a median follow-up period of 20.5 months after HSCT.

**Conclusions:**

MRD is critical for predicting de novo AML outcomes. Most MRD-positive patients cannot achieve MRD negativity with conventional chemotherapy. Thus, HSCT may be the primary therapeutic option for these patients.

## Introduction:

Despite the high remission rate (approaching 80% in younger adults) seen after intensive chemotherapy in patients with de novo acute myeloid leukemia (AML), only 30% to 40% of these patients survive for 5 years after diagnosis.^1^ Unfortunately, many patients experience relapse, probably due to the presence of minimal residual disease (MRD). Regarding the prognostic factors for post-remission relapse, a great effort has been made to detect and characterize MRD using various prognostic and therapeutic strategies. For example, specific gene rearrangements such as *PML/RARα*, *AML1/ETO*, and *CBFB/MYH11* have been used to represent MRD using nested reverse transcriptase-polymerase chain reaction (RT-PCR).^2^–^4^ However, this technique applicable in only 20% to 30% of AML patients.^5^ Furthermore, *AML1/ETO* or *CBFB/MYH11* transcripts are often detectable in patients who were considered to be cured of leukemia.^4^,^6^ Therefore, PCR for the detection of these transcripts after complete remission (CR) may be an equivocal tool because the significance of MRD positivity is undetermined, although MRD positivity on PCR closely correlates with a subsequent relapse in patients with the *PML/RARα* fusion gene.^2^,^3^ On the other hand, multiparameter flow cytometry (MFC) may be a useful method for MRD detection as it defines leukemia-associated phenotypes (LAP) in AML cells at diagnosis in almost all AML patients.^7^ Several reports have been published showing that MRD detection using MFC at post-induction/-consolidation is a valuable tool for predicting relapse.^8^–^15^ However, to our best knowledge, there is little information regarding the use of MRD detection by MFC in Japanese AML patients for the optimization of post-remission therapy. Therefore, we attempted to identify characteristic LAP from bone marrow specimens taken at diagnosis in individual patients using three color flow cytometry and assessed the relationship between LAP and clinical outcomes.

## Patients and Method:

### Eligibility:

A total of 33 consecutive adult de novo AML patients with LAP who were diagnosed between April 2006 and March 2009 and treated to accomplish a morphologic CR at the Department of Hematology and Clinical Immunology, Kobe City Medical Center General Hospital (Kobe, Japan) were analyzed. We experienced a total of 49 AML patients in the period; 40 with LAP and 9 without LAP. The achievement of a morphologic CR after inductinon chemotherapy was a criterion for inclusion in this study, while the exclusion criteria were AML without LAP; myelodysplastic syndrome (MDS)-derived overt AML; therapy-related AML; and *PML/RARα*-associated AML (AML-M3), in which RT-PCR has been reported to be a most valuable tool as described above [2,3]. Approval for this study was obtained from the institutional review board.

### Treatment Protocols:

As the induction chemotherapy, combined cytarabine (100 mg/m^2^/day, days 1–7) and daunorubicin (45 mg/m^2^/day, days 1–3) were administered. In the case of induction failure, re-induction chemotherapy composed of enocitabine (200 mg/m^2^/day, days 1–7), mitoxantrone (8 mg/m^2^/day, days 1–3), and etoposide (100 mg/m^2^/day, days 1–5) was performed. For the refractory cases, which could not achieve a hematologic CR after 2 courses of induction therapy, salvage regimens depended on the physicians’ choice. As consolidation chemotherapy, the patients received high-dose cytarabine (6 g/m^2^/day, days 1–3) for 4 cycles. When the patients were more than 70 years old, had impaired kidney, heart, lung, or liver function, the dosage of chemotherapeutic agents was reduced 30% to 50% when considered necessary by the physicians. We performed allogeneic hematopoietic stem cell transplantation (allo-HSCT) for suitable cases, which were chosen according to the Japan Society for Hematopoietic Cell Transplantation (JSHCT) guidelines (JSHCT monograph Vol. 6: http://www.jshct.com/guidline/, The Japan Society for Hematopoietic Cell Transplantation Web).

### Detection of LAP and MRD:

At presentation, immunophenotypic studies using 3-color flow cytometry were performed to detect LAP in bone marrow specimens. AML cells were identified using a CD45/side scatter gating strategy, and LAP were detected by staining leukemia cells with a triple fluorescence assay, which consisted of staining for CD45 and that for other two antigens, including myeloid/monocytic markers (CD11b, 13, 14, 33, 36, 86, 117), lymphoid markers (CD2, 4, 7, 19, 56), and miscellaneous markers (CD34, HLA-DR),^7^ as shown in **table 1**. A given combination of markers was regarded as relevant if it was expressed in at least 50% of blasts. Two or more antibody combinations, when possible, were employed to determine the LAP for each patient. The CellQuest software (Becton Dickinson, Mountain View, CA) was used for MFC data acquisition.^16^ MRD studies during CR were performed on erythrocyte-lysed whole bone marrow cells using the same antibody combination as used for each LAP at diagnosis. To abstract myeloblast group efficiently, instead of erythroblasts, monocytes, or promyelocytes, we adopted allophycocyanin (APC)-conjugated anti-CD34 and phycoerythrin-Cy7 (PE-Cy7)-conjugated anti-CD45 monoclonal antibodies gating strategy after CD45/side scatter gating. We determined a cell group as MRD only when the group was definitely different from its normal counterpart in the expression of antigens described as above and comprised more than 0.10% of total nucleated cells.

### Statistical analysis:

Overall survival (OS) and relapse free survival (RFS) were calculated from the date on which we first accomplished morphologic CR. CR, relapse, and RFS were defined by standardized criteria.^17^ The Kaplan-Meier method was used to estimate OS and RFS. For comparisons of OS and RFS between two groups, the log-rank test was applied. To evaluate the independent effects of different variables on the duration of OS and RFS, multivariate analysis was performed, using the Cox proportional hazards model with predictive variables that were significant in the univariate analysis. The other statistical tests included t tests, χ^2^ tests, and Fisher’s exact tests. All calculations were made using the program JMP 8.0 (SAS Institute, Cary, NC, US). All P values of <0.05 were considered to be significant.

## Results:

### Clinical Characteristics of AML Patient:

The clinical characteristics of 33 AML patients are shown in **table 2**. We classified the patients into Group A (20 patients, MRD positive) and B (13 patients, MRD negative), according to the detection of MRD by MFC at the first hematologic CR. The median follow up period was 16 months (4–42 months). Between Group A and B, there was no significant difference regarding age, sex predominance, French-American-British (FAB) subtype, white blood cell (WBC) count, cytogenetic risk, performance status before treatment, or the proportion of patients in which less than 50% of AML cells were positive for myeloperoxidase (MPO), although the number of patients who required 2 or more remission induction therapies to achieve hematologic CR was 14 in Group A and 2 in Group B (*P*=.0067). All of these factors other than sex were previously suggested as unfavorable prognostic factors in Japanese patients.^18^ We were obliged to reduce the treatment intensity for 9 and 5 patients in Group A and B, respectively. Allogeneic stem-cell transplantation (allo-SCT) was performed in 6 patients of Group A and 6 of Group B, without statistical significance. Seven of 9 AML patients without LAP achieved morphological CR within 2 courses of chemotherapy, with 2-year OS rate of 55.6%. On the other hand, 33 of 40 patients with LAP accomplished a morphologic CR, with 2-year OS rate of 63.0%.

### Impact of MRD After CR Induction:

**Figure 1.**
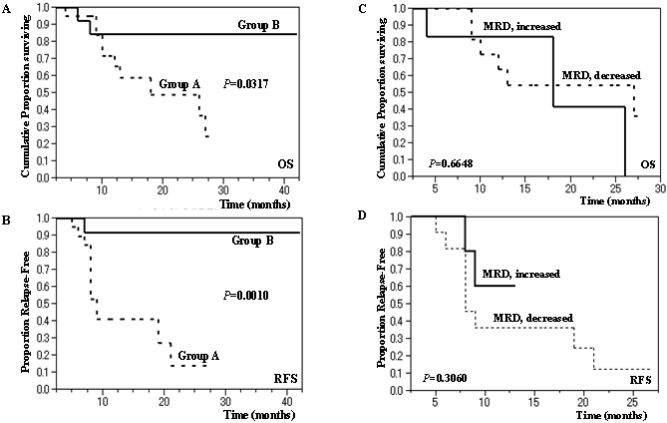
Overall survival (A) and relapse free survival (B) of AML patients according to MRD-positivity after induction chemotherapy. Overall survival (C) and relapse free survival (D) of AML patients according to MRD fluctuation during the initial consolidation therapy.

As shown in **figure 1**, Group A exhibited a significantly lower survival rate than Group B (*P*=.0317) with 2-year OS rates of 49.0% and 84.6%, respectively. Notably, in Group A, the patients who underwent single induction therapy were found to be more likely to show better survival than those who required 2 or more rounds of induction therapy (*P*=.1475) with 2-year OS rates of 75.0% and 39.4% respectively. Moreover, Group A showed much worse RFS than Group B (*P*=.0010) with 2-year RFS rates of 13.7% and 91.7%, respectively.

### Impact of MRD Fluctuation After CR Induction:

We divided Group A into 2 subgroups: ‘MRD increased (11 patients)’ and ‘MRD decreased (6 patients)’ according to the effects of the first consolidation chemotherapy administered when the patient was in a morphologic CR state. The remaining patients consisted of one patient who relapsed after the first post-induction therapy and two who had a nearly stable MRD level. The level of MRD at postinduction and postconsolidation (3 courses) was 0.10 to 1.28% with a median value of 0.60% and 0.25 to 3.44% with a median value of 1.04% among ‘MRD increased’ group, respectively; 0.18 to 1.75% with a median value of 0.32% and 0.00 to 0.47% with a median value of 0.14% among ‘MRD decreased’ group, respectively. As shown in **figure 1** (C,D), there was no difference between the 2 subgroups with regard to OS or RFS (*P*=.6648 and *P*=.3060, respectively). Of note, intensive consolidation chemotherapy eradicated MRD in only 2 of 20 cases in Group A.

### Impact of Prognostic Factors on OS and RFS:

As shown in **table 3**, several significant factors were highlighted by univariate analysis, which led to the conclusion that OS was correlated with MRD positivity at post-induction, 2 or more courses of required induction therapy, and 3 or more PS at diagnosis (*P*=.0249, *P*=.0367, and *P*=.0370, respectively) and that RFS was related to MRD positivity (*P*=.0005). Although we found relatively higher cytogenetic risk and lower WBC count in GroupA compared to those of Group B in **table 2**, these variables did not exhibit any significance in univariate analysis. All of these relevant prognostic variables were subjected to a multivariate model. In this analysis (**table 4**), MRD-positivity and a high level of PS (3 or more) were found to be independent variables significantly associated with a shorter duration of RFS and OS, respectively (*P*=.0042 and *P*=.0400, respectively).

## Discussion:

The MRD studies reported in the literature indicate that 60% to 94% of AML patients have an aberrant phenotype of AML cells, as evaluated by MFC at diagnosis,^7^,^19^ which are much higher frequencies than those demonstrated by PCR detection of specific gene rearrangements such as *PML/RARα*, *AML1/ETO*, and *CBFB/MYH11*. LAP have traditionally been classified into three groups: cross-lineage expression of lymphoid antigens on AML cells; asynchronous expression of antigens, for example, when blasts express both immature and mature antigens; and under-expression or over-expression of antigens.^5^ In our analysis, a total of 75 LAP were identified; 25 cross-lineage expressions, 10 asynchronous expressions, and 40 under-expressions. Unlike PCR, in which sensitivity is affected by variations in the expression levels of the target genes in AML cells, MFC directly measures cell number and can be combined with cell sorting of selected cell populations for further characterization.^5^ To increase the sensitivity of LAP detection, the exploitation of 6–8 color antibody panels and uncommon markers such as CD15 and CD65 may have a positive effect.^7^

Regarding the impact of MRD positivity on clinical outcomes, several reports have been published providing evidence that MRD studies using MFC are valuable tools for predicting relapse.^8^–^15^ In our study, it was shown that MRD detection by 3-color flow cytometry after successful remission induction therapy had a great impact on the OS and RFS of patients with AML. Therefore, information regarding MRD positivity is very useful for physicians for evaluating clinical outcomes in relation to the necessity of allo-SCT at a relatively early stage of the clinical course.^15^ Notably, only a few patients in Group A obtained MRD negativity in the present study, even after several courses of intensive consolidation chemotherapy strongly suggesting that Group A type patients require allo-SCT because of their high risk of early relapse. This concept is supported by the fact that 4 patients of Group A who underwent allo-HSCT when in the CR state were still alive without relapse at a median follow-up period of 20.5 months after HSCT, while 2 patients who necessarily received allo-SCT during the chemotherapy-refractory state died 2 or 3 months after the transplantation. In our cohort, however, we need to take into account that we analyzed the data from relatively limited number of patients and that the result might be confused by the potential effect of allo-HSCT. Therefore, in the future, it may be reasonable that we determine the indication of allo-HSCT based on LAP-determined MRD in a larger cohort of patients. Similarly, although we found the fact that MRD fluctuation during consolidation therapy has no significant impact on the prognosis as shown in **Figure 1** (C,D), the accumulation of cases should be needed. Although Al-Mawali et al. and San Miguel et al. demonstrated a correlation between the relapse rate and the levels of MRD after induction,^20^,^21^ Venditti et al. found that the level of MRD after consolidation was the most important prognostic factor.^13^ The differences in the therapeutic induction regimens between these studies may explain the contradictory results; the formers adopted less intensive therapy for induction, as employed in the present study. We also observed a reduction in the importance of MRD positivity after 3 courses of consolidation therapy regarding OS and RFS (*P*=.1864 and *P*=.0025, respectively) compared with MRD positivity after induction. In previous reports, the threshold discriminating MRD-negative from MRD-positive cases was set at 0.15% to 1% at postinduction^9^,^20^,^21^ and 0.035% to 0.2% at postconsolidation.^9^,^15^,^19^,^21^ Given that 3-color flow cytometry has the ability to achieve a sensitivity of 0.1% to 0.01% (one leukemic cell in 1,000 to 10,000 nucleated cells),^19^,^22^ we considered whether the detection of MRD by even 3-color flow cytometry is a reliable marker for predicting the outcome of AML patients who have achieved hematologic CR. In fact, the level of MRD at postinduction in the present study was 0.10 to 1.75% with a median value of 0.28%. In patients with the *AML1/ETO* or *CBFB/MYH11* fusion gene, we examined bone marrow samples using RT-PCR in addition to MFC. RT-PCR exhibited higher sensitivity; all 13 samples (3 patients) without LAP-associated MRD turned out to be positive for *AML1/ETO* or *CBFB/MYH11* fusion transcript. However, 2 of 3 patients (one with *AML1/ETO* and another with *CBFB/MYH11*) survived without relapse in spite of prolonged PCR-positivity at a follow-up period of 26 and 13 months, respectively, suggesting that LAP-associated MRD is a more valuable tool for predicting prognosis, although the third case subsequently suffered a relapse.

Nevertheless, we have some problems with the specificity and sensitivity of MFC as a diagnostic tool for MRD detection. For example, LAP may undergo phenotypic shifts,^7^ which result in false negativity. Also, considering the specificity of LAP, we cannot deny the possibility that the normal counterpart of AML cells may have identical of similar antigen profiles. To overcome these defects, some reports have suggested the feasibility of the real-time quantitative PCR (RQ-PCR) assay for detecting *nucleophosmin* (*NPM1*) mutations and Wilms’ Tumor gene (*WT1*) overexpression as well as established genetic markers including *PML/RARα*.^23^–^27^ Although WT1 is overexpressed in more than 70% of AML cases,^28^ the levels of *WT1* expression in normal bone marrow cells are relatively high.^27^ Thus, low levels of *WT1* may not be useful for distinguishing MRD from the background levels of *WT1* expression derived from normal hematopoietic cells. On the other hand, *NPM1* mutations are observed exclusively in leukemic cells, being identified in approximately 35% of de novo AML and in 50% of AML cases with a normal karyotype.^29^ We have recently established nested RQ-PCR assays for detecting MRD by the use of *NPM1* mutations using patient-specific primers, which yield assay sensitivities of 0.0001%. Thus, RQ-PCR is considered a more valuable method than MFC especially in cases with *NPM1* mutation. These techniques may be useful for detecting MRD as a means of predicting clinical outcomes.

## Conclusions:

MRD detected by 3-color flow cytometry at a relatively early stage of treatment may provide a lot of valuable information to physicians concerning the risk of AML recurrence and indications for allo-HSCT. The accumulation of studies employing 6-or-more color MFC and PCR assays for novel gene targets is necessary to establish more precise optimization of postremission therapy for AML patients.

## Figures and Tables

**Table 1.** The combination of immunophenotyping at diagnosis. (FITC, fluorescein isothiocyanate; PE, phycoerythrin; PE-Cy7, Phycoerythrin-Cy7; cyMPO, cytoplasmic myeloperoxidase).

**FITC**	**PE**	**PE-Cy7**
CD4	CD19	CD45
CD7	CD33	CD45
CD2	CD13	CD45
HLA-DR	CD11b	CD45
CD36	CD14	CD45
CD34	CD56	CD45
CD86	CD117	CD45
cyMPO	cyCD79a	CD45

**Table 2.** Clinical Characteristics of the Patients.

**Characteristics**	**Group A (n=20)**	**Group B (n=13)**
Age, years, median (range)	60.5(17–73)	55(38–71)
Sex (Male/Female), No. of patients	9/11	7/6
FAB subtype, No. of patients	M1(3), M2(9)	M1(1), M2(4)
	M4(2), M5(6)	M4(2), M5(6)
WBC count, /μL, median (range)	8,100(700–161,400)	34,100(600–340,000)
Cytogenetic risk*, No. of patients Favorable/Intermediate/Unfavorable	3/17/0	4/9/0
Relapse cases at diagnosis, No. of patients	2	3
PS at diagnosis ≧ 3, No. of patients	3	4
MPO < 50% at diagnosis, No. of patients	4	5
Required induction therapy ≧ 2, No. of patients	14	2
Allo-SCT, No. of patients	6	6

* Patients were stratified, according to the Medical Research Council classification of cytogenetic risk. (FAB, French-American-British; WBC, white blood cell; PS, performance status; MPO, myeloperoxidase; Allo-HSCT, allogeneic hematopoietic stem cell transplantation).

**Table 3.** Impact of Prognostic Factor on OS and RFS according to Univariate Analysis.

	**OS**		**RFS**	

	Hazard Ratio (95% CI)	*P*	Hazard Ratio (95% CI)	*P*
MRD (+) at postinduction	4.65 (1.19–30.67)	0.0249	14.03 (2.65–260.24)	0.0005
WBC > 20,000μL	0.96 (0.30–3.07)	0.9413	0.96 (0.33–2.81)	0.9343
MPO < 50% at diagnosis	0.39 (0.06–1.49)	0.1840	0.52 (0.12–1.68)	0.2940
Age > 50 year old	0.99 (0.31–3.71)	0.9822	1.77 (0.54–7.87)	0.3634
Required induction therapy ≧ 2	3.52 (1.08–13.50)	0.0367	2.68 (0.92–8.82)	0.0717
PS at diagnosis ≧ 3	3.72 (1.09–11.78)	0.0370	3.05 (0.93–8.91)	0.0644
Cytogenetic risk other than favorable*	0.52 (0.16–1.95)	0.3064	0.71 (0.22–3.15)	0.6155

OS, overall survival; RFS, relapse free survival; MRD, minimal residual disease; WBC, white blood cell; MPO, myeloperoxidase; PS, performance status

* Patients were stratified, according to the Medical Research Council classification of cytogenetic risk.

**Table 4.** Impact of Prognostic Factor on OS and RFS according to Multivariate Analysis.

	OS		RFS	

	Hazard Ratio (95% CI)	*P*	Hazard Ratio (95% CI)	*P*
MRD (+) at postinduction	2.97 (0.56–22.75)	0.2076	13.18 (2.05–263.13)	0.0042
Required induction therapy ≧ 2	2.26 (0.60–10.70)	0.2374	1.13 (0.37–3.97)	0.8338
PS at diagnosis ≧ 3	3.74 (1.07–12.45)	0.0400	2.87 (0.86–8.73)	0.0840

OS, overall survival; RFS, relapse free survival; MRD, minimal residual disease; PS, performance status.
